# Phytotherapy in Pediatric Dentistry: A Narrative Review of Clinical Applications and Evidence

**DOI:** 10.3390/children12111559

**Published:** 2025-11-17

**Authors:** Zorela Elena Miclăuș, Rahela Tabita Moca, Ruxandra-Ilinca Matei, Abel Emanuel Moca, Adriana Țenț, Anca Porumb

**Affiliations:** 1 Doctoral School of Biomedical Sciences, University of Oradea, 1 Universității Street, 410087 Oradea, Romania; zore_miclaus@yahoo.com; 2 Department of Dentistry, Faculty of Medicine and Pharmacy, University of Oradea, 4 Universității Street, 410087 Oradea, Romania; rahelamoca@gmail.com (R.T.M.); abelmoca@uoradea.ro (A.E.M.); adriana.tent@uoradea.ro (A.Ț.); anca.porumb@uoradea.ro (A.P.)

**Keywords:** phytotherapy, pediatric dentistry, herbal medicine, oral health

## Abstract

**Background/Objectives:** Phytotherapy, the use of plant-derived bioactive compounds for therapeutic purposes, has gained increasing attention in dentistry as a natural, well-tolerated, and culturally acceptable adjunct to conventional treatments. In pediatric dentistry, its potential relevance lies in its antimicrobial, anti-inflammatory, and antioxidant properties, which may support oral health, caries prevention, pulp vitality, and gingival health. This narrative review aimed to summarize the current clinical evidence regarding the application of phytotherapeutic agents in pediatric oral care. **Methods**: A narrative review was conducted according to SANRA guidelines, including clinical studies on plant-based products used for preventive or therapeutic purposes in children and adolescents. **Results**: Forty-three clinical studies met the inclusion criteria. The most commonly investigated agents included licorice, green tea, cocoa husk, cranberry, pomegranate, *Aloe vera*, and miswak. These agents demonstrated antimicrobial activity against cariogenic bacteria, reduction in plaque and gingival indices, and favorable healing in pulp therapies. In endodontics, *Aloe vera*-derived acemannan and Ankaferd Blood Stopper^®^ showed outcomes comparable to conventional materials, while pomegranate and apple cider vinegar exhibited partial antibacterial effects as irrigants. **Conclusions**: Phytotherapy shows promise as a complementary approach in pediatric dentistry, contributing to caries prevention, gingivitis control, and pulp healing. However, current evidence remains limited by small sample sizes, short-term follow-ups, and heterogeneity in formulations. Further trials are required to confirm efficacy, ensure safety, and standardize phytotherapeutic applications in pediatric oral care.

## 1. Introduction

Oral diseases such as dental caries, gingivitis, periodontitis, and mucositis are common conditions in children and adolescents, affecting quality of life, nutrition, speech, and self-esteem [1,2]. According to the World Health Organization, tooth decay remains the most prevalent noncommunicable disease and a major global public health concern, with children and adolescents being significantly affected [3].

Phytotherapy is a branch of complementary medicine that uses medicinal plants and plant extracts for therapeutic purposes. It relies on natural active compounds, such as flavonoids, tannins, essential oils, alkaloids, and mucilages, derived from various plant parts (roots, leaves, flowers, bark, resins) to prevent or treat different conditions [4,5,6]. Unlike conventional medicine, which isolates single active molecules, phytotherapy harnesses the synergistic action of multiple bioactive substances within a plant. This approach is considered gentler and less invasive, an important aspect in pediatric treatments.

However, this synergistic bioactivity also presents both potential advantages and inherent challenges in clinical application. The presence of multiple active molecules may enhance therapeutic efficacy through additive or complementary mechanisms but simultaneously introduces challenges in terms of standardization, bioavailability, and reproducibility [7,8]. In contrast to single-compound agents such as chlorhexidine or fluoride, herbal extracts often exhibit variable concentrations and pharmacokinetics, making their clinical effects less predictable [8]. These translational limitations underline the importance of rigorous formulation control, dose optimization, and comparative clinical testing when integrating phytotherapy into evidence-based pediatric dentistry [9].

Owing to its antimicrobial, anti-inflammatory, antioxidant, and tissue-regenerative properties, phytotherapy has emerged as a promising option for oral care, particularly in pediatric populations [10].

The oral cavity of children presents specific characteristics compared with that of adults: the oral microbiome is still developing and more prone to dysbiosis; the local immune system is immature; plaque-induced gingivitis is common, especially during adolescence, due to hormonal changes [11,12,13]. Moreover, children’s oral tissues are more reactive and sensitive, requiring therapeutic agents that are biocompatible, well tolerated, and pleasant-tasting [14]. Within this context, phytotherapeutic agents may offer advantages through their natural antimicrobial, anti-inflammatory, and antioxidant actions, along with their generally favorable tolerability and sensory acceptability [15].

Oral therapies must, therefore, be safe, well tolerated, and age appropriate. Phytotherapy can meet these requirements, particularly in topical formulations (gels, mouthrinses, toothpastes, sprays), as it is generally well tolerated, has few side effects, and is often more acceptable to parents [16,17]. Phytotherapy does not replace standard dental treatments (toothbrushing, fluoride applications, restorations, etc.), but it can serve as a valuable adjunct in the prevention and management of mild oral diseases. As an adjunctive approach, phytotherapeutic formulations may enhance the effectiveness of conventional treatments by reducing microbial load and gingival inflammation, promoting tissue repair, and improving local healing after procedures such as scaling, pulpotomy, or minor surgery [18]. In some contexts, adjunctive phytotherapy may also contribute to lowering the frequency of professional interventions or the need for systemic drug use, particularly antibiotics or anti-inflammatory agents, which is highly relevant in pediatric care [19]. It may improve patient comfort, reduce the need for systemic drug therapies, and promote local healing. At the same time, it is essential that phytotherapeutic products be tested, properly formulated, and used under professional supervision. However, despite the increasing availability of herbal oral care products, scientific evidence supporting their efficacy and safety in pediatric dentistry remains limited and fragmented. This underscores the need for a comprehensive synthesis of current data on their potential therapeutic roles.

The aim of this narrative review is to summarize the current evidence regarding the use of plant-derived extracts (phytoderivatives) in oral therapy for children and adolescents.

## 2. Materials and Methods

### 2.1. Search Strategy

A narrative review was conducted to identify and summarize current clinical evidence regarding the use of plant-based or herbal products in pediatric dentistry. The search aimed to explore the preventive and therapeutic potential of phytotherapeutic agents in common oral conditions among children, such as dental caries, gingivitis, and endodontic infections.

Relevant studies were retrieved from PubMed, Web of Science and Scopus databases, covering the period January 1995 to September 2025. The review followed the methodological recommendations of the Scale for the Assessment of Narrative Review Articles (SANRA) to ensure transparency, quality, and comprehensiveness.

The following Boolean search strategy was applied: (“phytotherapy” OR “herbal medicine” OR “plant extract”) AND (“pediatric dentistry” OR “paediatric dentistry” OR “oral health” OR “oral hygiene” OR “dental caries” OR “tooth decay” OR “gingivitis” OR “periodontal disease” OR “pulpotomy” OR “endodontic treatment”) AND (child OR children OR adolescent OR infant OR toddler).

Only English-language, full-text articles were considered. The literature search was performed between 10 September and 25 September 2025. Reference lists of eligible studies and related reviews were also screened to identify additional relevant publications.

### 2.2. Study Selection and Eligibility Criteria

Two authors independently conducted the literature search and screened all records by title and abstract, selecting those most relevant to the objectives of this narrative review. All records retrieved from the three databases were exported into EndNote 20.2. (Clarivate Analytics, Philadelphia, PA, USA) for reference management and automatic duplicate removal. The deduplicated library was subsequently imported into Rayyan (Qatar Computing Research Institute, Doha, Qatar) to facilitate blinded and structured screening. Both reviewers independently performed title and abstract screening, followed by full-text assessment of potentially eligible studies. Discrepancies were resolved by discussion and consensus.

The inclusion and exclusion criteria were established to ensure that the review focused on original clinical research providing empirical evidence on the use of phytotherapeutic or plant-based products in pediatric dentistry.

Studies were included if they met the following criteria:

- Original clinical research (randomized or non-randomized trials, pilot studies, or observational studies);
- Studies involving children or adolescents up to 18 years of age;
- Studies that evaluated herbal or plant-derived agents used for preventive or therapeutic purposes in oral conditions such as dental caries, gingivitis, pulp therapy, or other oral diseases;
- Articles published between January 1995 and September 2025;
- Studies written in English and available in full-text format.

Studies were excluded if they met any of the following conditions:

- Animal studies, systematic reviews, or narrative commentaries;
- Articles not directly involving a pediatric population;
- Studies investigating products without a clear phytotherapeutic component;
- Grey literature, including theses, conference abstracts, or non–peer-reviewed sources.

The initial search identified 512 articles from PubMed (*n* = 224) and Web of Science (*n* = 288). After removing 174 duplicates, 338 unique records remained. Following title and abstract screening, 238 articles were excluded for not meeting inclusion criteria. A total of 100 full-text articles were assessed for eligibility, of which 57 were excluded (non-clinical studies, adult populations, or insufficient data). Ultimately, 43 studies met all inclusion criteria and were included in this narrative review.

For each study meeting the inclusion criteria, data were manually extracted using a predefined standardized template developed by the authors. Extracted variables included author, year of publication, country, study design, population characteristics, type and formulation of the phytotherapeutic product, target oral condition, primary outcomes, and main conclusions. Two authors independently verified the extracted data to ensure accuracy and consistency. The included studies (*n* = 43) were subsequently organized into three thematic domains corresponding to the main areas of pediatric dental practice: Dental Caries and Oral Biofilm Control, Application in Pediatric Endodontic Treatments, and Use in Gingivitis and Other Pediatric Oral Conditions. This thematic categorization guided the structure of the Narrative Synthesis section.

**Figure 1:**
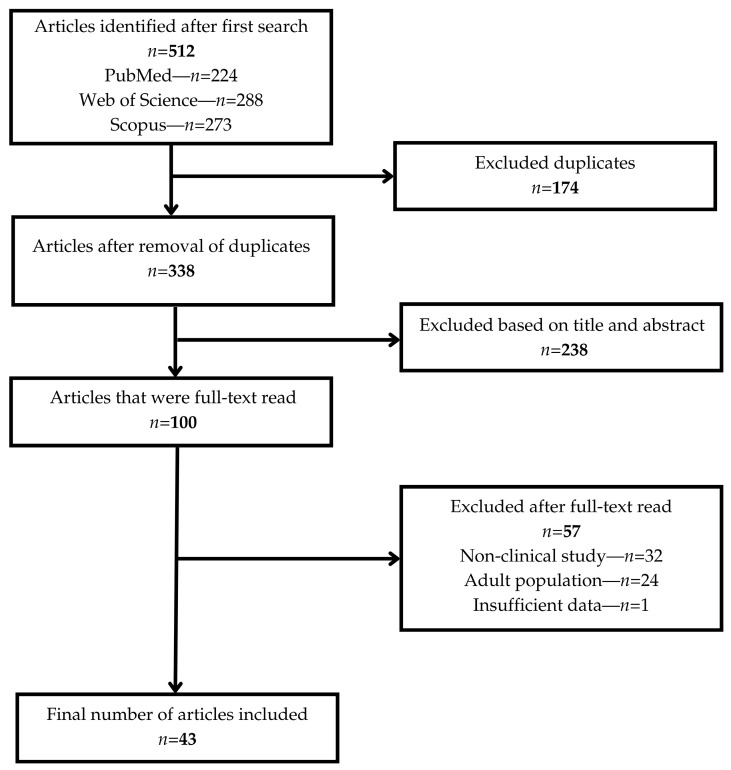
PRISMA flow diagram illustrating the study selection process.

The study selection process is illustrated in Figure 1, presented in a PRISMA-style flowchart.

## 3. Narrative Synthesis

### 3.1. Dental Caries and Oral Biofilm Control

#### 3.1.1. Epidemiological Context and Etiological Factors

Dental caries in permanent teeth remains the most prevalent disease worldwide, with an age-standardized prevalence of 27,500 cases per 100,000 population, affecting approximately 2.24 billion individuals. In Europe, the prevalence is comparable, reaching 28,700 cases per 100,000 population [20]. Early childhood caries (ECC), which affects the primary dentition in children under six years of age, shows a highly variable prevalence which is influenced by multiple factors such as race, culture, ethnicity, socioeconomic status, and oral hygiene practices that differ across countries. Consequently, reported prevalence rates range widely from 1% to 85.5% [21].

This variability is closely linked to the etiological factors of dental caries. The interaction between the tooth surface, dietary sugars, bacterial plaque, and the duration of exposure is fundamental to the initiation and progression of dental caries [22]. In ECC, early sugar introduction, bottle-feeding, and nocturnal feeding, particularly breastfeeding beyond 12 months of age, are dietary habits that can accelerate the development of carious lesions [23]. Dietary control and the reduction of sugar intake are therefore essential components of caries prevention [24].

Dental plaque also plays a crucial role in the onset and rapid progression of carious lesions. Although physiologically present on tooth surfaces, plaque is involved in the etiology of both dental caries and periodontal disease, conditions associated with alterations in the microbial balance of the oral biofilm [25,26]. The microorganisms that most commonly implicated in the caries process include *Streptococcus mutans*, other acidogenic streptococci, *Rothia* spp., *Actinomyces* spp., *Lactobacillus* spp., *Candida albicans*, and *Selenomonas sputigena*. Among these, *S. mutans* is one of the most significant due to its ability to metabolize a wide range of carbohydrates and to adapt to abrupt ecological changes within the biofilm [27].

Caries prevention therefore requires not only dietary management but also effective plaque control. Mechanical oral hygiene remains the primary method of plaque removal, supported by adjunctive measures such as dental floss, interdental brushes, and mouthrinses [28]. In addition to conventional treatments, phytotherapy has been increasingly explored as an adjuvant approach to oral hygiene, aiming to control bacterial plaque and reduce *Streptococcus mutans* levels [29].

#### 3.1.2. *Glycyrrhiza uralensis* (Licorice Root)

Given the well-documented preference of children and adolescents for sweet foods [30], several strategies have been developed to replace sugar in commonly consumed products, making them healthier alternatives. One approach involves the use of sugar-free lollipops containing *Glycyrrhiza uralensis* (licorice root) extract as an adjunctive preventive measure against dental caries in children.

A pilot study conducted in the United States among preschoolers showed that consuming these lollipops twice daily for three weeks significantly reduced salivary *Streptococcus mutans* levels in children at high caries risk. The reduction persisted for up to 22 days after discontinuation, although bacterial counts gradually returned toward baseline thereafter [31]. Similar outcomes were reported in a randomized, double-blind clinical trial in Turkey involving 108 children with and without caries risk: significant reductions in *S. mutans* were observed only among high-risk children who had not previously undergone dental treatment, suggesting that the lollipops may play a complementary role in cases of low treatment compliance [32].

Additionally, a study conducted in China among high-risk preschoolers confirmed the efficacy of licorice root lollipops, demonstrating an over 80% reduction in *S. mutans* levels after three weeks, while maintaining or even enhancing the diversity of the oral microbiome, indicating a targeted antimicrobial action against cariogenic bacteria without disrupting the overall microbial balance [33].

The effectiveness of *Glycyrrhiza uralensis* extract has also been demonstrated through other delivery forms. Mouthrinses containing aqueous or ethanolic licorice extracts have been shown to immediately decrease *S. mutans* counts and increase salivary pH in children [34], while ethanolic gel formulations exhibited comparable efficacy to chlorhexidine solutions in reducing cariogenic bacteria [35].

Collectively, these findings support the potential of licorice root extract as a promising phytotherapeutic agent for caries prevention in children, with favorable safety and acceptability profiles.

#### 3.1.3. *Camellia sinensis* (Green Tea, EGCG)

Green tea, derived from the leaves of *Camellia sinensis*, is one of the most widely consumed beverages worldwide and contains numerous bioactive compounds [36]. The literature highlights multiple health-promoting properties of green tea, including antimetastatic, anticancer, hepatoprotective, antidiabetic, anti-obesity, and anti-atherosclerotic effects [37]. In recent years, its potential benefits for oral health have gained increasing attention within the dental research community [38].

In pediatric dentistry, Vilela et al. [39] valuated the effects of a purified green tea extract, epigallocatechin-3-gallate (EGCG), in comparison with plain green tea, chlorhexidine, and distilled water on salivary levels of cariogenic bacteria in children aged 5–12 years with high caries risk. Following a single one-minute rinse, both green tea and EGCG significantly reduced *Streptococcus mutans* and *Lactobacillus* spp. colony counts. EGCG exhibited stronger antibacterial activity than green tea, achieving mean reductions of 79.9% for *S. mutans* and 72.1% for *Lactobacillus* spp., compared to 68.3% and 59.2% for green tea, respectively; chlorhexidine achieved 95.5% and 92.3% reductions, while distilled water achieved 50.6% [39]. The authors concluded that, although chlorhexidine remains the most potent antimicrobial agent, green tea and EGCG represent safer, well-tolerated alternatives for children’s oral hygiene, with EGCG offering additional benefits by inhibiting bacterial glucosyltransferases and reducing plaque acidogenicity.

These findings are consistent with earlier research by Ferrazzano et al. (2011), who demonstrated that a one-week use of green tea extract significantly reduced *S. mutans* and *Lactobacillus* spp. levels in adolescents (12–18 years old) compared with placebo, confirming its in vivo antimicrobial potential [40]. Similarly, Hegde and Kamath showed that a 0.5% green tea mouthrinse was as effective as a chlorhexidine-fluoride combination and only slightly less effective than chlorhexidine alone in reducing salivary *S. mutans* and *Lactobacillus* spp. levels in children aged 8–12 years [41]. More recent evidence from Kamath et al. further confirmed that a 0.5% green tea extract performed comparably to 0.12% chlorhexidine in reducing *S. mutans* colonies in dental plaque, supporting the hypothesis that green tea represents a promising natural alternative for plaque control and caries prevention in children [42].

#### 3.1.4. *Theobroma cacao* (Cocoa Husk)

The husk of the cocoa bean (*Theobroma cacao*) contains numerous bioactive compounds with antibacterial and glucosyltransferase-inhibiting properties, making it a potential agent for plaque control [43,44]. Matsumoto et al. [44] reported that cocoa bean husk (CBH) extract inhibited the in vitro adhesion of *Streptococcus mutans* to hydroxyapatite and reduced the number of viable bacteria in dental plaque samples collected from children aged 4–15 years. In the same study, in vivo use of CBH mouthrinses for four consecutive days, without other oral hygiene procedures, significantly reduced plaque index scores and salivary *S. mutans* levels [44].

These findings were later corroborated by a 2008 clinical trial involving 32 children who used a mouthrinse containing cocoa husk extract. Compared with placebo, the participants exhibited a 20.9% mean reduction in *S. mutans* counts and a 49.6% decrease in plaque index, confirming the extract’s antimicrobial and antiplaque effects [45]. More recently, Fajriani et al. (2016) demonstrated that a single rinse with ethanolic cocoa husk extract in children aged 12–14 years led to a rapid and substantial decline in salivary *S. mutans* colonies, from 59.1 CFU/mL to 9.4 CFU/mL within just 30 min, indicating an immediate and pronounced antibacterial action [46].

#### 3.1.5. *Vaccinium macrocarpon* (Cranberry)

Cranberry (*Vaccinium macrocarpon*) initially attracted the attention of the scientific community for its beneficial effects on urinary tract health [47]. Later, in the 21st century, its phytochemical compounds were investigated for their potential roles in cancer prevention and vascular protection [48]. The biological properties of cranberry include inhibitory effects on bacterial adhesion, biofilm formation, and microbial growth [49], which has led to increasing interest in its application for dental plaque control.

An in vitro study conducted in 2019 on oral biofilms derived from children demonstrated that cranberry extracts significantly reduced the acidogenicity and metabolic activity of *Streptococcus mutans* and *Candida albicans*. Moreover, the biofilm structure became disorganized, and the number of colonies of both species decreased [50]. Clinically, a randomized trial involving preschool children with active caries evaluated the effectiveness of tablets combining an inactivated paraprobiotic (*Ligilactobacillus salivarius* CECT 5317) with cranberry extract rich in proanthocyanidins. Daily administration for three months resulted, after nine months of follow-up, in a 44% overall reduction in caries incidence compared to placebo. Although no significant differences were found in salivary *S. mutans* or *Lactobacillus* spp. levels or in oral hygiene indices, the study highlighted the potential of paraprobiotic–polyphenol combinations as a preventive strategy for children at high caries risk [51]. Other clinical studies have also confirmed that cranberry-based mouthrinses reduce *S. mutans* colonies in children [52].

#### 3.1.6. *Punica granatum* (Pomegranate)

The pomegranate (*Punica granatum* L.) is a fruit native to arid and semi-arid regions and belongs to the family *Punicaceae*, whose only genus is *Punica* [53]. All parts of the plant, seeds, juice, peel, and root, can be used for medicinal purposes, being rich sources of punicic acid, polyphenols, and alkaloids [53]. The health benefits of pomegranate have been documented in inflammatory, cardiovascular, metabolic, and oncologic conditions [54].

Regarding its application in dental plaque control among children, a recent study demonstrated that hydroalcoholic extract obtained from the whole fruit possesses antibacterial activity against *Streptococcus mutans* and *Lactobacillus acidophilus*, though to a lesser extent than chlorhexidine. Clinically, however, a mouthrinse containing 38% pomegranate extract significantly reduced dental plaque in children aged 8–10 years, achieving an OHI-S reduction of 34% compared to 36% for chlorhexidine, with no statistically significant difference between the two [55].

Additionally, Mishra et al. [56] compared the effects of *P. granatum*, *Terminalia chebula*, and *Vitis vinifera* extracts in children and found that all three significantly reduced *S. mutans* colonies. Among them, pomegranate showed the highest efficacy and superior substantivity, maintaining its antimicrobial effect even after discontinuation [56].

These results support the use of pomegranate as a viable phytotherapeutic alternative for dental plaque control in children, offering comparable effects to conventional agents but with fewer adverse reactions.

#### 3.1.7. *Aloe vera*

*Aloe vera* has been traditionally used for topical treatment of various skin lesions [50]. This perennial plant, characterized by its green leaves and tubular yellow flowers, has been extensively investigated for its therapeutic properties, including anti-inflammatory, anticancer, antidiabetic, antioxidant, and antimicrobial effects [57].

Concerning its role in oral biofilm control, a pilot study involving adolescents with intellectual disabilities compared an *Aloe vera*-based toothpaste with a triclosan-containing formulation. After 30 days of use, the *Aloe vera* group showed a significantly greater reduction in plaque index (−44.3% vs. −28%) and gingival index (−45.6% vs. −27.4%), as well as a marked decrease in oral *Candida albicans* colonies [58].

These findings suggest that *Aloe vera* may serve as a safe and effective alternative to conventional antimicrobial agents for maintaining oral health in children and adolescents, particularly among vulnerable populations.

#### 3.1.8. *Ocimum sanctum* (Tulsi, Holy Basil)

*Ocimum sanctum* L., commonly known as Tulsi or “Holy Basil,” is a perennial aromatic herb from the family *Lamiaceae*, native to India and Southeast Asia [59]. Used for centuries in Ayurvedic medicine and considered sacred in Hindu tradition, Tulsi contains a wide array of bioactive compounds, such as eugenol, carvacrol, ursolic acid, and flavonoids, that contribute to its anti-inflammatory, antioxidant, and antimicrobial properties [60].

In dentistry, research has primarily focused on its effects against cariogenic bacteria, particularly *Streptococcus mutans* [61]. A clinical study conducted in children aged 9–12 years evaluated the effects of chewing Tulsi leaves on salivary pH and bacterial viability. While no significant changes were observed in salivary pH, *S. mutans* colony counts decreased substantially, confirming the plant’s direct antimicrobial action on cariogenic bacteria [62].

Furthermore, a randomized trial compared a 4% ethanolic Tulsi mouthrinse with 0.05% sodium fluoride and *Terminalia chebula* extract. After seven days of use, all three groups exhibited significant increases in salivary pH and reductions in *S. mutans* colonies. However, the Tulsi extract produced the most rapid reduction (within one hour), suggesting a fast-acting and promising antimicrobial effect as a phytotherapeutic adjunct in caries prevention [63].

#### 3.1.9. Other Plant Extracts

In addition to the most extensively studied plants for caries prevention in children, several other herbal extracts have been clinically evaluated, yielding promising results. For instance, a herbal mouthrinse demonstrated efficacy comparable to chlorhexidine in reducing dental plaque and oral *Streptococcus* counts, outperforming liquid probiotic formulations [64]. Another polyherbal preparation showed similar outcomes to chlorhexidine in maintaining oral health among schoolchildren over a three-month period, significantly decreasing plaque and gingival indices [65].

Moreover, mouthrinses containing extracts of mango (*Mangifera indica*) and mint (*Mentha arvensis*, also known as pudina) have been found to reduce salivary *Streptococcus mutans* levels in children. Notably, mint extract was also effective against *Candida albicans*, producing results comparable to those of chlorhexidine [66]. Additional studies investigated the topical application of olive oil, alone or in combination with white turmeric (*Curcuma zedoaria*) and neem (*Azadirachta indica*), and reported significant reductions in dental plaque and oral bacterial load in hospitalized children, with the most pronounced effects observed for the combination with white turmeric [67].

Furthermore, the daily consumption of a traditional herbal tea prepared from tsaang-gubat (*wild tea*) over a 17-month period reduced caries incidence by more than 75% among schoolchildren, although the benefits gradually diminished once regular consumption ceased [68]. In addition, dental wipes impregnated with *Triphala* extracts were shown to significantly decrease salivary *Streptococcus mutans* colonies in children with intellectual disabilities, providing a practical option for patients unable to perform effective toothbrushing [69].

Overall, current evidence indicates that, alongside conventional dietary and oral hygiene measures, a range of plant-derived extracts exhibit noteworthy antimicrobial and antiplaque effects in children. These botanicals have been tested in various formulations, such as lollipops, mouthrinses, oral gels, toothpastes, tablets, and traditional teas, demonstrating variable but generally favorable efficacy profiles combined with good safety and tolerability. Although their effectiveness may not always match that of chlorhexidine, these plant-based agents represent promising and complementary strategies for the prevention of dental caries and control of oral biofilm in pediatric populations (Table 1).

### 3.2. Application in Pediatric Endodontic Treatments

#### 3.2.1. Direct Pulp Capping in Primary Teeth

If left untreated, caries in primary teeth may progress to the pulp chamber, leading to pulpal inflammation and endodontic complications [70,71]. The most common complications include pulpitis and acute apical periodontitis [72]. These conditions require endodontic intervention, ranging from direct pulp capping to pulpectomy, depending on several clinical factors [73]. The structural particularities of primary teeth, together with the complex microbial flora associated with endodontic infections, necessitate the use of antimicrobial agents and materials with high efficacy and proven biocompatibility [66]. However, conventional agents such as formocresol or calcium hydroxide present several limitations, including cytotoxicity, incomplete antimicrobial action, or unpredictable resorption, which has fueled the search for phytotherapeutic alternatives [74,75].

Direct pulp capping in primary teeth is indicated only when the tooth is vital, the pulpal exposure is less than 1 mm, and occurs during cavity preparation or because of trauma. Additionally, bleeding should be fresh and easily controlled [76]. The materials most commonly used for direct pulp capping are calcium hydroxide and mineral trioxide aggregate (MTA) [76], as well as Biodentine [77]. Although these materials show variable success rates, challenges related to application technique, adhesion issues, and high costs may limit their routine use in primary teeth [78].

In the pursuit of alternative materials, a study published in 2016 compared the six-month clinical, radiographic, and histological outcomes of pulps treated with acemannan versus calcium hydroxide when used as direct pulp capping agents in deeply carious primary teeth approaching exfoliation [79]. This was, in fact, the only study identified that evaluated a phytotherapeutic product for direct pulp capping in primary teeth. Acemannan is a naturally derived polysaccharide extracted from *Aloe vera*, characterized by high biocompatibility, and has demonstrated clinical performance comparable to calcium hydroxide [80]. The study by Songsiripradubboon et al. (2016) showed that acemannan used as a direct pulp capping material in deeply carious primary teeth produced clinical and radiographic outcomes similar to those of calcium hydroxide, but exhibited superior histological results, with reduced inflammation and more frequent dentin bridge formation [79].

#### 3.2.2. Pulpotomy in Primary Teeth

The use of phytotherapeutic agents in pulpotomy procedures has been somewhat better documented. Pulpotomy is a conservative endodontic approach involving the partial or total removal of the coronal pulp tissue [81]. This technique is widely used and recommended for primary teeth with deep carious lesions or reversible, and even irreversible, pulpal involvement [81,82]. The materials employed for pulpotomy in primary teeth include mineral trioxide aggregate (MTA), Biodentine, formocresol, and ferric sulfate [83]. Each of these agents presents specific advantages and limitations, and the search for an optimal pulpotomy material continues. Within this context, four studies have been identified that investigated the effects of phytotherapeutic products in primary tooth pulpotomy.

Three of these studies evaluated Ankaferd, a hemostatic agent traditionally used for centuries in the Anatolian region. Ankaferd is a combination of five plant extracts, *Glycyrrhiza glabra*, *Thymus vulgaris*, *Alpinia officinarum*, *Vitis vinifera*, and *Urtica dioica*, which together provide rapid hemostasis and have an expanding range of clinical indications [84]. Building on these properties, Ankaferd has been tested as a hemostatic agent in pulpotomy procedures.

The first study, published in 2012, assessed the clinical and radiographic efficacy of Ankaferd Blood Stopper^®^ (ABS) compared with formocresol in 60 primary molars of 30 healthy children. The results demonstrated a 100% clinical and radiographic success rate at three months for both materials, with similar rates at six months (96.7% for formocresol and 93.3% for ABS) and a slight decline at 12 months (89.3% and 85.7%, respectively). The differences were not statistically significant, suggesting that ABS may represent a viable phytotherapeutic alternative to conventional pulpotomy agents, with comparable short- and medium-term outcomes [85].

A subsequent study by Özmen and Bayrak [86] compared ABS, formocresol, and ferric sulfate in 45 primary molars from 26 children aged 6–9 years, followed for up to 24 months. At the end of the follow-up period, clinical success rates were 87% for ABS, 87% for formocresol, and 100% for ferric sulfate, while radiographic success rates were 87%, 80%, and 87%, respectively. The differences were not statistically significant, and internal resorption was the most common cause of failure. The authors concluded that ABS demonstrates comparable efficacy to conventional pulpotomy materials, confirming its potential as a phytotherapeutic alternative [86].

The most recent study, published in 2025, compared ABS, ferric sulfate, and the Er,Cr:YSGG laser (BIOLASE Inc., Foothill Ranch, USA) in primary molar pulpotomies. Clinical success rates were high across all groups (100% for ABS and laser, 96% for ferric sulfate), whereas radiographic success was highest for the laser (96%), followed by ferric sulfate (76%) and ABS (73%). The authors concluded that although laser therapy achieved the most favorable overall outcomes, ABS exhibited comparable clinical efficacy, supporting its potential as a safe alternative in primary tooth pulpotomy [87].

A single study investigated the use of other phytotherapeutic agents in pulpotomy, comparing *Aloe vera* and *Nigella sativa* extracts with formocresol. The results showed that *Aloe vera* achieved superior clinical success (90.9% at 12 months) and comparable radiographic outcomes to formocresol (72.7% vs. 81.8%), confirming its promise as an alternative pulpotomy medicament. Conversely, *Nigella sativa* showed substantially lower clinical (40%) and radiographic (20%) success rates, which does not support its use in primary tooth pulpotomy [88].

#### 3.2.3. Root Canal Irrigation and Disinfection

In conventional endodontic treatment of primary teeth, sodium hypochlorite remains the irrigant of choice for canal disinfection [82]. However, a randomized clinical trial published in 2025 evaluated the antimicrobial efficacy of 5% pomegranate peel extract (PPE) and 5% apple cider vinegar (ACV) compared with 5% sodium hypochlorite (NaOCl) in disinfecting necrotic root canals of primary teeth. The study included 45 necrotic primary incisors from 30 children aged 3–5 years. All groups exhibited significant bacterial reduction: PPE achieved a mean reduction of 60.4% (3.38 log_10_ CFU/mL), ACV 51.4% (2.15 log_10_ CFU/mL), and NaOCl 87.5% (4.23 log_10_ CFU/mL). Although NaOCl remained the most effective irrigant, both PPE and ACV demonstrated relevant antibacterial activity and acceptable cytotoxicity profiles, suggesting their potential as natural alternatives in pediatric endodontic treatment, pending confirmation through long-term studies [89].

Current evidence regarding the use of phytotherapeutic agents in pediatric endodontics indicates that such products may serve as promising alternatives to conventional materials, achieving comparable clinical outcomes in certain contexts. *Aloe vera*-derived acemannan has shown superior histological results in direct pulp capping; Ankaferd Blood Stopper^®^ demonstrated comparable efficacy to formocresol and ferric sulfate in pulpotomy; and both pomegranate peel extract and apple cider vinegar exhibited notable antibacterial activity when used as irrigating agents.

Nevertheless, persistent limitations, such as the occurrence of internal resorption, the lower efficacy of certain extracts, and the scarcity of long-term clinical trials and comprehensive histological evaluations, underscore the need for further research before these agents can be widely recommended in pediatric endodontics (Table 2).

### 3.3. Use in Gingivitis and Other Pediatric Oral Conditions

#### 3.3.1. Prevention and Treatment of Gingivitis

Gingivitis is an inflammatory condition of the gingival tissues, caused by bacterial infection. Although gingivitis does not involve attachment loss, if left untreated it may progress to more severe forms of periodontitis [90]. Differences in the composition of the oral microbiome and the disruption of microbial homeostasis, caused by a variety of environmental and host-related factors, can exacerbate gingival inflammation and contribute to disease progression [26,91]. The prevalence of gingivitis among children and adolescents varies depending on the population studied and environmental influences, reaching up to 99% in certain groups [92]. Improving oral hygiene remains the cornerstone of both prevention and treatment, and it plays a critical role in preventing periodontal disease [93].

Several studies have evaluated the use of phytotherapeutic preparations in the control of dental plaque and gingival inflammation for the prevention and management of gingivitis. Among the most extensively studied is Triphala, a traditional Ayurvedic formulation composed of three fruits, *Terminalia chebula*, *Terminalia bellirica*, and *Phyllanthus emblica*, known for their antimicrobial and antioxidant properties. Two clinical trials (Bhattacharjee et al., 2014 [93]; Chainani et al., 2014 [94]) demonstrated that Triphala-based mouthrinses effectively reduced plaque accumulation and gingival inflammation in children and adolescents, with results comparable to those achieved using chlorhexidine but without its common side effects, such as tooth staining and taste alteration.

Promising results have also been reported for *Salvadora persica* (miswak), a plant traditionally used for oral hygiene in Arab and Asian regions. Amoian et al. [95] found that chewing gum containing *S. persica* extract significantly decreased gingival inflammation and bleeding on probing, even in the absence of professional scaling, suggesting a beneficial local action on gingival tissues [95].

Similarly, *Mangifera indica* (mango) has shown favorable effects on oral health. In a clinical study by Bhat et al. [96], a mouthrinse prepared from mango leaves significantly reduced plaque and gingival indices, as well as salivary *Streptococcus mutans* levels, achieving efficacy comparable to chlorhexidine [96].

These findings support the potential of phytotherapy as an effective and safe adjunct in the prevention and treatment of gingivitis in children, offering natural, accessible, and well-tolerated alternatives for maintaining oral health.

#### 3.3.2. Other Pediatric Oral Conditions

Another area of interest in pediatric phytotherapy involves its use in oral mucosal diseases and other non-cariogenic conditions, such as aphthous ulcers, herpes labialis and halitosis. A study by Dai et al. [97] investigated the use of Traditional Chinese Medicine (TCM) remedies for the management of oral conditions among Chinese-American pediatric populations. The survey included 318 families, of whom 45.6% of parents and 19.1% of children reported using TCM for dental or oral health problems. The most common indications were gingivitis and/or periodontitis (27.1%), halitosis (26.3%), aphthous ulcers (26.3%), toothache (25.2%), and herpes labialis (9.7%). The most frequently used phytotherapeutic products included watermelon frost (38.6%), niuhuang jiedu pian (17.3%), propolis, and honey (9.9%). The study revealed that the use of such remedies was influenced by demographic and cultural factors, including parental age, language, child’s birthplace, and duration of residence in the United States, highlighting the cultural determinants underlying the adoption of natural therapies. The authors concluded that although these remedies are widely used within Asian communities, further studies are needed to scientifically validate their efficacy and safety in pediatric dental practice [97].

Another study relevant to the application of phytotherapy in pediatric dentistry was conducted by Kabil, Badran, and Wassel [98], who evaluated the effect of incorporating chlorhexidine and *Salvadora persica* (miswak) extract into conventional glass ionomer cement (GIC) on its clinical performance and antibacterial properties. The randomized clinical trial included 60 young permanent molars with deep carious lesions in children aged 6–9 years. While no significant differences were observed among the groups at three months, at six and nine months the clinical success rates were higher for the miswak group (100% and 90%, respectively) compared to chlorhexidine (75% and 60%). All groups demonstrated significant reductions in *S. mutans* counts in residual dentin. The addition of miswak extract improved the antibacterial properties of the restorative material without compromising its stability, suggesting its potential application in minimally invasive pediatric dentistry as a natural alternative to conventional antimicrobial agents [98].

The use of phytotherapeutic products in the management of gingivitis and other pediatric oral conditions highlights a substantial therapeutic potential, owing to the demonstrated antimicrobial, anti-inflammatory, and antioxidant effects of numerous plant-derived extracts. Preparations such as Triphala, *Salvadora persica*, and *Mangifera indica* have shown efficacy comparable to conventional agents but with better tolerability and fewer side effects. Nevertheless, further randomized controlled trials with larger sample sizes and standardized methodologies are needed to validate these findings and support their integration into pediatric dental practice (Table 3).

To provide a comprehensive overview, Table 4 summarizes the phytotherapeutic agents discussed in this review, highlighting their main microbial targets and clinical applications in pediatric dentistry.

## 4. Discussion

**Figure 2:**
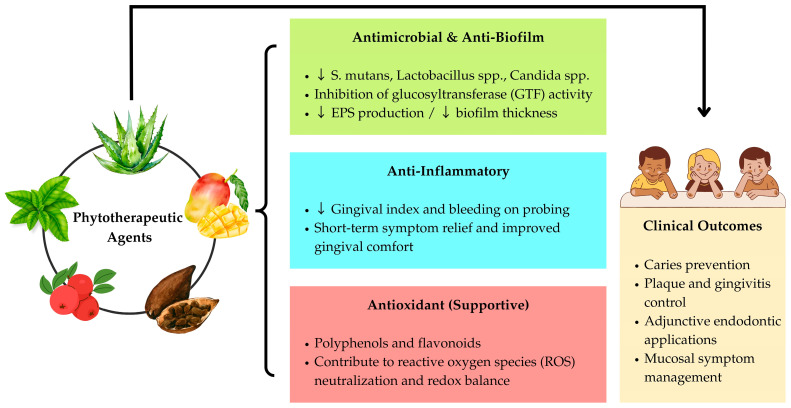
Mechanisms of action and clinical outcomes of phytotherapeutic agents in pediatric oral diseases.

This review highlights that several phytotherapeutic preparations, particularly extracts from *Glycyrrhiza uralensis* (licorice root) [31,32,33,34,35], *Camellia sinensis* (green tea/EGCG) [39,40,41,42], *Theobroma cacao* (cocoa husk) [44,45,46], *Vaccinium macrocarpon* (cranberry) [50,51,52], *Punica granatum* (pomegranate) [55,56], and Aloe vera [57], can reduce cariogenic bacterial load and plaque indices in children when used in various pharmaceutical forms (lollipops, mouthrinses, gels, toothpastes, tablets, and herbal teas). In pediatric endodontics, acemannan has shown promising results for direct pulp capping [79,80], Ankaferd Blood Stopper^®^ has demonstrated comparable performance to conventional materials in pulpotomy [84,85], and pomegranate peel extract and apple cider vinegar have exhibited antibacterial activity as irrigating agents, although less potent than sodium hypochlorite [89]. For gingivitis, formulations containing Triphala, *Salvadora persica* (miswak), and *Mangifera indica* have reduced plaque accumulation and gingival inflammation with good tolerability [93,94,95,96] (Figure 2).

From a pediatric dentistry perspective, these findings confirm the potential usefulness of phytotherapy as an adjunct to standard preventive measures, health education, dietary sugar control, twice-daily brushing with fluoridated toothpaste, and interdental cleaning [99]. The observed benefits are mainly reflected in reduced *S. mutans* and *Lactobacillus* spp. counts, lower plaque and gingival indices, and rapid improvements in some short-term studies. However, most studies rely on surrogate outcomes (e.g., salivary CFU counts, plaque or gingival indices) assessed over short follow-up periods, and few report robust clinical endpoints such as medium-term caries incidence. Therefore, these products should be recommended only as complementary options, not as replacements for the current standard of care. When comparing the clinical performance of phytotherapeutic products with that of single-compound agents, such as chlorhexidine or fluoride, it becomes evident that herbal formulations typically exhibit lower short-term efficacy but a more favorable tolerability and sensory profile. This difference reflects the multifactorial nature of plant-derived preparations, where multiple bioactive constituents act synergistically rather than through a single dominant pathway. While this polypharmacological profile may confer broader biological coverage (antimicrobial, anti-inflammatory, and antioxidant effects), it also introduces variability in bioavailability, standardization, and comparability across studies [7,8,15].

In the prevention of dental caries in children, the gold standard remains twice-daily toothbrushing with fluoridated toothpaste (≥1000–1450 ppm fluoride), dietary counseling to reduce the frequency of free sugar intake, professional fluoride applications for high-risk patients, and the use of fissure sealants on susceptible surfaces [99]. Within this framework, botanical extracts may be considered useful adjuncts when chlorhexidine is poorly tolerated or contraindicated for prolonged use due to side effects such as staining or dysgeusia, particularly in short-term programs aimed at improving oral hygiene [100]. However, in line with current guidelines, these agents cannot replace fluoride-based measures or behavioral interventions. Their integration into preventive practice requires standardized formulations and careful monitoring of commercial product composition.

In pediatric endodontics, NaOCl remains the reference irrigant [101], while calcium hydroxide and bioceramic materials (MTA, Biodentine) continue to represent the benchmark for pulp protection [102,103]. Available evidence suggests that *Aloe vera*-derived acemannan exhibits favorable histological outcomes in direct pulp capping of primary teeth, but its indication remains narrow and requires long-term validation [79,80]. Ankaferd Blood Stopper^®^ provides effective hemostasis and short- to medium-term clinical and radiographic results comparable to established agents, although questions remain regarding long-term persistence and the risk of internal resorption [94,95]. For canal irrigation, natural extracts such as pomegranate peel or apple cider vinegar can reduce bacterial load but do not match the efficacy of NaOCl; thus, they may be considered only as contextual alternatives (e.g., in cases of parental preference or contraindications), with full disclosure to families regarding the current level of evidence [89].

In gingivitis management, clinical guidelines emphasize mechanical plaque control through proper brushing techniques, the use of interdental brushes in adolescents, and professional scaling or prophylaxis when indicated [104]. Within this context, phytotherapeutic preparations such as Triphala, *Salvadora persica* (miswak), and *Mangifera indica* may be used short-term as adjuncts to standard hygiene regimens, especially when cultural preferences or tolerability issues lead to avoidance of chlorhexidine [93,94,95,96]. Their integration should be individualized, with attention to potential allergies, alcohol content, and the fact that many reported benefits originate from short-term studies relying on surrogate endpoints. Furthermore, phytotherapeutic agents may be particularly beneficial for children with special healthcare needs, who often experience greater challenges in maintaining oral hygiene due to sensory sensitivities or behavioral limitations [105]. The mild taste, pleasant aroma, and natural texture of some herbal formulations, such as Triphala toothwipes or mouthrinses, can enhance acceptance and compliance in this group [17,69]. These characteristics make phytotherapy a potentially inclusive adjunct for preventive oral care in pediatric populations with limited tolerance for conventional products.

The main limitations of the current evidence base include small sample sizes, short study durations, and high heterogeneity among plant extracts and pharmaceutical formulations, which hinder the comparability of results. Moreover, standardization of composition and assessment of long-term safety remain insufficient, and few studies report tangible clinical outcomes rather than microbiological parameters alone.

The biological mechanisms underlying the effects of phytotherapeutic compounds further support their potential role in pediatric oral care. Many plant-derived agents exhibit broad-spectrum antibacterial, antifungal, and anti-inflammatory actions, while others demonstrate antioxidant and biofilm-modulating effects that can influence microbial adhesion and virulence. Importantly, the oral microbiome in children is not limited to bacterial species but also includes viral and fungal communities, which interact dynamically and contribute to both oral health and disease pathogenesis [106]. The multimodal activity of phytotherapeutic compounds could therefore promote microbial balance by targeting multiple components of this complex ecosystem. Such agents may help reduce dysbiosis and support homeostasis within the developing pediatric oral microbiome, offering a biologically integrated and well-tolerated approach for both preventive and therapeutic purposes.

A major strength of this review lies in its integrated approach, encompassing the three core domains of pediatric dentistry, prevention, endodontics, and gingivitis, and its alignment with current professional guidelines, emphasizing the adjunctive role of natural phytotherapeutic products. To strengthen the evidence base, future research should focus on multicenter randomized clinical trials, standardization of plant extracts, and long-term evaluation of safety and efficacy, enabling the rational and predictable integration of phytotherapy into modern pediatric dental practice. In addition, future studies should also assess the tolerability and effectiveness of different phytotherapeutic formulations across distinct pediatric age groups. Children at various developmental stages exhibit differences in oral microbiological composition, taste perception, compliance, and behavioral responses, all of which may influence treatment outcomes [107,108]. Evaluating age-specific acceptability and adherence could therefore provide valuable insights for optimizing the formulation and delivery of plant-based products in pediatric oral care.

## 5. Conclusions

Phytotherapy shows promise as an adjunct in pediatric dentistry, supported by evidence of antimicrobial, anti-inflammatory, and antioxidant effects. Extracts from *Glycyrrhiza uralensis*, *Camellia sinensis*, *Theobroma cacao*, *Vaccinium macrocarpon*, *Punica granatum*, *Aloe vera*, and *Salvadora persica* have demonstrated benefits in reducing cariogenic bacteria, controlling plaque and gingivitis, and promoting pulp healing.

Although encouraging, current data are limited by small samples and short-term designs; thus, these natural agents should be regarded as complementary to standard preventive and therapeutic measures. Further well-designed clinical trials and standardized formulations are essential to enable their safe and evidence-based integration into pediatric oral care.

## Figures and Tables

**Table 1:** Summary of studies evaluating phytotherapeutic agents for caries prevention and oral microbial control in children.

Author (Year, Country)	Design/Sample	Phytotherapeutic Agent (Formulation)	Main Outcomes/Conclusions
Peters et al., 2010 (USA) [31]	Pilot interventional study, preschool children	*Glycyrrhiza uralensis* (licorice) lollipop, twice daily for 3 weeks	Significant reduction in salivary S. mutans counts, especially in high-caries-risk group; effect lasted ~3 weeks post-intervention.
Almaz et al., 2017 (Turkey) [32]	Randomized controlled trial, 108 children (5–11 years)	Sugar-free herbal lollipop containing licorice extract (10 days)	Marked reduction in S. mutans levels in high-risk children; safe and acceptable preventive adjunct.
Chen et al., 2019 (China) [33]	Controlled clinical study, 37 preschool children (3–6 years)	*Glycyrrhiza uralensis* lollipop (glycyrrhizol A) twice daily for 3 weeks	>80% reduction in S. mutans; preserved microbial diversity; well tolerated.
Jain et al., 2013 (India) [34]	Double-blind pilot trial, 60 children (7–14 years)	Aqueous (15%) and ethanolic (3.75%) licorice mouthrinses	Both extracts significantly reduced S. mutans and increased salivary pH; ethanolic form more effective and palatable.
Kumar et al., 2020 (India) [35]	In vivo comparative study, 30 schoolchildren (6–12 years)	Aqueous and ethanolic licorice gels (1.75 g/10 mL and 350 mg/10 mL)	Ethanolic extract showed greater antibacterial activity, comparable to 0.2% CHX; suitable for pediatric use.
Vilela et al., 2020 (Brazil) [39]	Randomized clinical trial, 80 children (6–12 years)	Camellia sinensis tea/epigallocatechin gallate (EGCG) mouthrinse	Both formulations significantly reduced S. mutans counts vs. control; no adverse effects reported.
Ferrazzano et al., 2011 (Italy) [40]	Randomized controlled in vivo study, 66 adolescents (12–18 years)	Green tea extract mouthrinse, 3×/day for 7 days	Significant reduction in S. mutans and Lactobacillus; confirmed antimicrobial efficacy of green tea.
Kamath et al., 2021 (India) [42]	Randomized controlled trial, 50 children (8–12 years)	0.5% green tea mouthrinse vs. 0.12% chlorhexidine	Comparable reduction in S. mutans; green tea better tolerated.
Matsumoto et al., 2004 (Japan) [44]	Experimental (in vitro and in vivo), 19 children	Theobroma cacao bean husk extract (mouthrinse/chewing gum)	Inhibited glucosyltransferase and reduced plaque formation in vivo.
Srikanth et al., 2008 (India) [45]	Single-blind crossover study, 32 children (10–14 years)	Cocoa bean husk extract (1 mg/mL mouthrinse)	Significant 49.6% reduction in S. mutans and 20.9% in plaque accumulation (*p* < 0.001).
Fajriani et al., 2016 (Indonesia) [46]	Time-series experimental study, 30 children (12–14 years)	Cocoa bean husk ethanol extract (0.1% mouthrinse)	Significant short-term S. mutans reduction (*p* < 0.05); effective antibacterial effect.
Philip et al., 2019 (Australia/UK) [50]	In vitro dual-species biofilm model	*Vaccinium macrocarpon* (cranberry polyphenol extract)	Inhibited S. mutans–Candida albicans virulence and acidogenicity; reduced EPS and biofilm thickness.
Olczak-Kowalczyk et al., 2025 (Poland/Denmark) [51]	Double-blind RCT, 73 preschool children	Paraprobiotic (*Ligilactobacillus salivarius*) + cranberry extract tablets	9-month follow-up showed reduced early carious lesions (ICDAS 1–2); safe and well tolerated.
Bansal et al., 2024 (India) [52]	Double-blind randomized controlled trial, 280 children (8–12 years)	Non-dialyzable cranberry mouthrinse vs. sodium fluoride rinse	Cranberry rinse non-inferior to fluoride; significant S. mutans reduction and good acceptance.
Mahd et al., 2023 (Iran) [55]	Double-blind crossover clinical trial + in vitro phase, 14 children (8–10 years)	Hydroalcoholic extract of whole *Punica granatum* fruit (38% mouthwash)	Significant inhibitory effect on S. mutans and Lactobacillus acidophilus in vitro; clinically decreased plaque index by 34%—comparable to 0.12% CHX (36% reduction); well tolerated and safe.
Mishra et al., 2019 (India) [56]	Randomized double-blind clinical trial, 80 children (8–15 years)	*Punica granatum* mouthrinse vs. Terminalia chebula and Vitis vinifera	All reduced S. mutans; P. granatum showed highest substantivity and long-lasting antibacterial effect.
Khatri et al., 2017 (India) [58]	Double-blind RCT, 40 adolescents with intellectual disability	Aloe vera toothpaste vs. triclosan-based control	Significant reduction in plaque and gingival indices and Candida counts (*p* < 0.05).
Agarwal et al., 2010 (India) [61]	In vitro study	*Ocimum sanctum* (Tulsi) ethanolic extract (0.5–10%)	4% concentration produced 22 mm inhibition zone against S. mutans.
Lolayekar & Kadkhodayan, 2019 (India) [62]	Clinical study, 30 children (9–12 years)	Chewing fresh Tulsi leaves	Significant reduction in S. mutans colony counts; minimal effect on salivary pH.
Megalaa et al., 2018 (India) [63]	Randomized controlled trial, 60 children	*Ocimum sanctum* (Tulsi, 4%) and *Terminalia chebula* (2.5%) mouthrinses vs. 0.05% NaF	Increased salivary pH and decreased S. mutans counts; Tulsi more effective short-term, myrobalan better long-term.
Mishra et al., 2014 (India) [64]	Randomized controlled trial, 60 children (6–14 years)	Herbal oral rinse (Herboral) vs. 0.2% chlorhexidine and probiotic rinse	Herbal rinse equally effective as CHX in reducing S. viridans and plaque; better taste acceptance.
Kajjari et al., 2024 (India) [66]	Randomized controlled trial, 45 children (7–10 years)	*Mangifera indica* (mango) and Mentha arvensis (mint) mouthrinses vs. CHX (0.2%)	Both herbal rinses significantly reduced S. mutans and Candida albicans; Mentha an early equivalent to CHX.
Deshpande et al., 2025 (India) [67]	Triple-blinded randomized clinical trial, 84 hospitalized children (3–14 years)	Topical application of extra virgin olive oil (EVOO), EVOO + 35% *Curcuma zedoaria* (white turmeric), EVOO + 30% *Azadirachta indica* (neem) vs. saline	All preparations reduced plaque and bacterial counts at 72 h (*p* < 0.05). EVOO + CZ and EVOO + AI achieved 100% plaque reduction, 95–96% S. mutans and Lactobacillus decrease, and 75–90% Candida reduction; well tolerated and practical for hospitalized children.
Parajas, 1997 (Philippines) [68]	Longitudinal school-based intervention (~3 years)	Wild tea (Ehretia microphylla, “tsaang-gubat”) beverage	~75% decrease in caries incidence; protective effect diminished with irregular use; low-cost culturally accepted strategy.
Deshpande et al., 2021 (India) [69]	Randomized controlled trial, 27 intellectually disabled children	Triphala toothwipes vs. placebo	Significant reduction in S. mutans at 48 h and 7 days; effective adjunct for children with special needs.

**Table 2:** Summary of studies evaluating phytotherapeutic agents in pediatric endodontic applications.

Author (Year, Country)	Design/Sample	Procedure	Phytotherapeutic Agent (Formulation)	Main Outcomes/Conclusions
Songsiripradubboon et al., 2016 (Thailand) [79]	Randomized controlled clinical and histologic study, 42 teeth in 37 children (7–11 y)	Direct pulp capping in primary molars	Aloe vera–derived acemannan sponge (0.4%)	6-month clinical and radiographic success rates were 72.7% (acemannan) vs. 70.0% (Ca(OH)_2_). Histological findings showed superior dentin bridge formation and pulpal healing in the acemannan group. Acemannan proved biocompatible and is a promising alternative for vital pulp therapy.
Yaman et al., 2012 (Turkey) [85]	Randomized single-blind clinical study, 30 children (6–9 y), 60 molars	Pulpotomy	Ankaferd Blood Stopper^®^ (1:1 dilution, topical)	At 12 months, clinical success was 89.3% (FC) vs. 85.7% (ABS), with no statistically significant difference. ABS was found equally effective and may serve as a natural pulpotomy medicament.
Özmen & Bayrak, 2017 (Turkey) [86]	Randomized clinical study, 26 children (6–9 y), 45 molars	Pulpotomy	Ankaferd Blood Stopper^®^ (standard solution, 15 s)	After 24 months, clinical success rates were 87% (ABS, FC) and 100% (FS); radiographic success 87%, 80%, and 87%, respectively. ABS demonstrated comparable outcomes to FC and FS and was deemed a safe alternative.
Şahin et al., 2025 (Turkey) [87]	Randomized clinical trial, 65 children (5–9 y), 81 molars	Pulpotomy (hemostasis phase)	Herbal hemostatic mixture (*Thymus vulgaris*, *Vitis vinifera*, *Glycyrrhiza glabra*, *Alpinia officinarum*, *Urtica dioica*)	At 12 months, clinical success was 100% for herbal and laser groups, 96% for FS. Radiographic success: 73% (herbal), 76% (FS), 96% (laser). Herbal agent provided satisfactory hemostasis comparable to FS but lower radiographic results; laser performed best overall.
Sharaf et al., 2023 (Egypt) [88]	Triple-blind randomized controlled trial, 66 molars (4–7 y)	Pulpotomy	Aloe veraethanolic extract vs. Nigella sativa extract	Clinical success after 12 months: 90% (A. vera), 40% (N. sativa), 72.7% (FC). Radiographic success: 72.7%, 20%, and 81.8%, respectively. Aloe vera showed promising biocompatibility and antibacterial potential; N. sativa not recommended for pulpotomy.
Mando et al., 2025 (Syria) [89]	Double-blind randomized clinical trial, 30 children (3–5 y), 45 incisors	Root canal irrigation (necrotic teeth)	*Punica granatum* peel extract (5%) and apple cider vinegar (5%)	Bacterial reduction: PPE 60.4%, ACV 51.6%, NaOCl 87.5%. Cell viability: PPE 85%, ACV 79% (ISO 10993-5 compliant). Both natural irrigants were less effective than NaOCl but demonstrated antibacterial activity and acceptable biocompatibility, with PPE showing superior safety.

**Table 3:** Summary of studies on phytotherapeutic agents in gingivitis and other pediatric oral conditions.

Author (Year, Country)	Design/Sample	Condition	Phytotherapeutic Agent (Formulation)	Main Outcomes/Conclusions
Bhattacharjee et al., 2015 (India) [93]	Randomized double-blind controlled trial, 60 schoolchildren (mean 13 y)	Plaque-induced gingivitis	Triphala aqueous mouthrinse (10 mL, 30 s, twice daily for 15 days)	Both groups showed significant plaque and gingival index reduction (*p* < 0.001). Triphala was as effective as CHX and can be used as a cost-effective, well-tolerated short-term alternative.
Chainani et al., 2014 (India) [94]	Double-blind crossover RCT, 120 schoolchildren (13–16 y)	Plaque and gingivitis	*Triphala mouthrinse* (10%)	Both CHX and Triphala significantly reduced plaque and gingival indices vs. control (*p* < 0.001); no significant difference between Triphala and CHX. Confirms comparable antiplaque and anti gingivitis efficacy.
Amoian et al., 2010 (Iran) [95]	Double-masked randomized trial, 72 adolescents (mean 15 y)	Gingivitis (plaque-induced)	*Salvadora persica* (Miswak) extract chewing gum	Significant improvement in gingival and bleeding indices (GI, BI) at 7 and 14 days vs. control. Persica gum promoted periodontal health, effective especially post-scaling.
Bhat et al., 2017 (India) [96]	Clinical comparative study, 20 children (8–14 y)	Plaque, gingival inflammation, S. mutans reduction	*Mangifera indica* (mango) leaf mouthwash	Both rinses improved plaque and gingival scores with microbial reduction; CHX slightly superior. Mangifera indica is a safe, acceptable herbal alternative.
Dai et al., 2016 (USA) [97]	Cross-sectional parental interview study, 318 Chinese families (340 children < 12 y)	Use of herbal medicine for oral conditions	Traditional Chinese herbal agents (e.g., watermelon frost, Niuhuang jiedu pian, propolis, Yunnan baiyao)	19.1% of children and 45.6% of parents reported TCM use for oral conditions (mostly aphthous ulcers, gingivitis, halitosis). Use strongly associated with parental habits and cultural factors. Demonstrates wide use of TCM in pediatric populations.
Kabil et al., 2017 (Egypt) [98]	Randomized clinical trial, 60 young permanent molars (6–9 y)	Deep caries restoration/antibacterial GIC	GIC modified with aqueous *Salvadora persica* extract (100%) or 0.5% CHX	Both CHX- and Miswak-modified GIC showed enhanced antibacterial effect vs. control. Miswak addition yielded high restoration survival (90% at 9 mo) with good biocompatibility.

**Table 4:** Summary of phytotherapeutic agents, microbial targets, and dental applications in pediatric oral care.

Botanical Source	Main Microbial Targets	Main Clinical Applications
*Glycyrrhiza uralensis* (Licorice, glycyrrhizol A)	*S. mutans*, *Lactobacillus* spp.	Caries prevention, plaque reduction
*Camellia sinensis* (Green tea, EGCG)	*S. mutans*, *Lactobacillus* spp.	Caries prevention, plaque reduction
*Theobroma cacao* (Cocoa bean husk extract)	*S. mutans*	Caries prevention, plaque reduction
*Vaccinium macrocarpon* (Cranberry polyphenols)	*S. mutans*, *Candida albicans*	Caries prevention, plaque reduction
*Punica granatum* (Pomegranate peel extract)	*S. mutans*, *Lactobacillus acidophilus*	Caries prevention, plaque reduction, root canal irrigation
Aloe vera (Acemannan, anthraquinones)	*Candida albicans*, mixed flora	Plaque and gingivitis control, direct pulp capping, pulpotomy
*Ocimum sanctum* (Tulsi)	*S. mutans*, *Candida* spp.	Caries prevention, plaque reduction
Herbal oral rinse (Herboral) (polyherbal blend: *Glycyrrhiza*, *Terminalia chebula*, *Azadirachta indica*)	*S. viridans*, *S. mutans*	Caries prevention, plaque reduction
*Mangifera indica* (Mango leaf extract)	*S. mutans*, *Candida albicans*	Caries prevention, plaque reduction, gingivitis control
*Mentha arvensis* (Mint)	*S. mutans*, *Candida albicans*	Caries prevention, plaque reduction
*Olea europaea* (EVOO) + *Curcuma zedoaria* (white turmeric) + *Azadirachta indica* (neem)	*S. mutans*, *Lactobacillus* spp., *Candida* spp.	Caries prevention, plaque reduction, gingivitis control
*Ehretia microphylla* (“Tsaang-gubat”, wild tea)	*S. mutans*	Caries prevention, plaque reduction
Triphala (*Terminalia chebula*, *Emblica officinalis*, *T. bellirica*)	*S. mutans*, *Lactobacillus* spp.	Caries prevention, plaque reduction, gingivitis control
Ankaferd Blood Stopper^®^ (*Thymus vulgaris*, *Vitis vinifera*, *Glycyrrhiza glabra*, *Alpinia officinarum*, *Urtica dioica*)	Mixed oral bacteria (*S. mutans*, *Actinomyces* spp.)	Pulpotomy (vital pulp therapy, hemostasis)
*Salvadora persica* (Miswak extract)	*S. mutans*, *Actinomyces* spp., *Lactobacillus* spp.	Plaque control, gingivitis management, restorative material enhancement
Traditional Chinese herbal medicines (e.g., *Propolis*, *Niuhuang jiedu pian*, *Yunnan baiyao*)	Broad-spectrum antibacterial and antifungal action	Aphthous ulcer, gingivitis, halitosis management, oral mucosal care

## Data Availability

This article is a narrative review and does not report any new data. All data supporting the findings discussed are available from the cited literature.
